# Predictors of Loneliness and Psychological Distress in Older Adults During the COVID-19 Pandemic: National Cross-Sectional Study

**DOI:** 10.2196/78728

**Published:** 2025-11-14

**Authors:** Rahela Orlandini, Antonela Matana, Deana Švaljug, Ivana Gusar, Vesna Antičević

**Affiliations:** 1 Faculty of Health Sciences, University of Split, Ruđera Boškovića 35, Split, 21000, Croatia, 385 21564813; 2 School of Medicine, University of Split, Split, Croatia; 3 Faculty of Health Studies, University of Rijeka, Rijeka, Croatia; 4 Department of Health Studies, University of Zadar, Zadar, Croatia

**Keywords:** older adults, COVID-19, loneliness, psychological distress, preference for solitude, pandemic-specific stressors, self-efficacy

## Abstract

**Background:**

The COVID-19 pandemic has significantly affected the mental health of older adults, particularly through increased loneliness and psychological distress. While various contributing factors have been studied, the role of preference for solitude as a potential predictor and mediator remains poorly understood.

**Objective:**

This national cross-sectional study aimed to examine predictors of loneliness and psychological distress among older adults during the pandemic, with a specific focus on preference for solitude and its mediating role between pandemic-specific stressors and self-efficacy.

**Methods:**

A total of 2053 Croatian residents aged 65 years and older were recruited using snowball sampling. Validated instruments were used, including the UCLA Loneliness Scale, Preference for Solitude Scale, Clinical Outcomes in Routine Evaluation-Outcome Measure, General Self-Efficacy Scale, and Pandemic-Specific Stressors Questionnaire for Older Adults. Hierarchical regression and path analysis were used, with statistical significance set at *P*<.05.

**Results:**

For loneliness, the final model explained 30.7% of the variance, with a coefficient of determination (*R*²) of 0.307 and a root mean square error of 0.708 (*P*<.001). Significant predictors included marital status (eg, never married: B=0.390, *P*<.001; psychological problems: B=0.020, *P*<.001; functionality: B=−0.037, *P*<.001; and social distancing: B=0.014, *P*<.001). Preference for solitude was also a significant predictor of loneliness (B=0.011; *P*<.001). For psychological distress, the final model explained 30.8% of the variance (*R*²=0.308; root mean square error=14.872; *P*<.001). Self-efficacy emerged as the strongest negative predictor of distress (B=−1.066; *P*<.001), whereas preference for solitude was a positive predictor (B=2.403; *P*<.001). The variable “spending several hours alone per day” was associated with lower levels of distress (B=−3.509; *P*=.003), while “frequent or superficial interactions with acquaintances (eg, at least once a week)” were related to higher distress (B=4.321, *P*=.002). Path analysis revealed that both social distancing and exposure to infection had a significant direct effect on self-efficacy: negative in the case of social distancing (*β*=−.391; *P*<.001) and positive for exposure to infection (*β*=.386; *P*<.001). However, preference for solitude did not significantly mediate either relationship, as indicated by nonsignificant indirect effects (*β*=−.006; *P*=.09 and *β*=.002; *P*=.48, respectively).

**Conclusions:**

Psychological problems and reduced functionality emerged as the strongest predictors of loneliness among older adults during the pandemic. Self-efficacy was the most important protective factor against psychological distress. Although preference for solitude may have adaptive benefits, in the context of this study, it was associated with increased loneliness and distress during enforced isolation. These findings suggest that public health interventions should balance respect for individual preferences with the provision of active support for vulnerable populations during crises.

## Introduction

In today’s uncertain times, it is essential to continually improve the well-being of older adults by learning from past experiences, such as the COVID-19 pandemic.

During the COVID-19 pandemic, periods of social isolation highlighted how personal preference for solitude (PS) versus feelings of loneliness can impact mental health. Differentiating solitude from social isolation and loneliness is essential for studying psychological well-being in older adults. Solitude is the objective state of being alone, which can feel positive, negative, or mixed, while social isolation and loneliness are commonly associated with negative emotions. Social isolation is the objective lack of social connections, and loneliness is the subjective perception of insufficient social contact; both are associated with depression, anxiety, and stress (as cited by Peng et al [1]). This paper explores the effects of involuntary social isolation and solitude resulting from pandemic-related measures on loneliness and the mental health of older adults.

Loneliness and psychological distress are significant mental health concerns, particularly among older adults during global crises [2]. Due to social isolation during the COVID-19 lockdown, older adults experienced significantly higher levels of loneliness [3,4] and psychological distress [5] compared with the pre-COVID period. This is supported by studies showing substantial increases in measurement scores [3,4]. Parlapani et al [6] demonstrate that older adults are not uniformly vulnerable to psychological distress during COVID-19; however, their capacity to adapt to adversity varies considerably. This variation depends on social, economic, and individual factors, aligning with previous findings on predictors of loneliness and psychological distress during the pandemic. There is substantial evidence indicating similar factors contribute to both loneliness and psychological distress.

Regarding sociodemographic factors, some research found that being female was associated with higher rates of distress and loneliness [7-9], while others did not observe this trend [10,11]. Similarly, some studies have found that advanced age in older adults is associated with greater loneliness [12-14] and psychological distress [9], but other studies reported the opposite [8,15]. Being unpartnered and living alone were consistent risk factors for loneliness [14,16] and psychological distress [17], while environmental factors such as housing type and rurality remain less studied. For older adults in Croatia, factors contributing to loneliness and stress included social isolation, movement restrictions, and intensive media coverage of the pandemic [18]. A pilot study on Croatian citizens found that older adults reported less loneliness during the pandemic if they lived with others, were more emotionally stable, had fewer psychological problems and pandemic-specific stressors, and engaged in fewer risky behaviors [19]. This study is now being expanded to a representative national sample of all older Croatian adults. In addition to loneliness, where the same predictors from the pilot study were retained, researchers are also examining predictors of psychological distress, focusing on time spent alone during lockdown, PS, and self-efficacy. Research shows that lifestyle changes, such as increased isolation and reduced social activities, disrupted older adults’ routines and harmed their mental health. One study found that those confined for more than 50 days were more likely to experience depression, with fear of infection linked to greater confusion, fatigue, tension, and reduced vigor [20]. Social isolation has also been connected to a wide range of adverse outcomes in older adults, including lower quality of life and cognitive decline. Other negative effects include loss of muscle mass, multimorbidity, and increased disability [21].

Studies also highlight the role of self-efficacy and personal perceptions in shaping psychological distress among older adults during lockdowns, emphasizing the need for adaptive coping strategies to support mental health. One study found that self-efficacy in the general population shaped coping mechanisms and distress levels during the initial lockdown [22]. A longitudinal study in Spain showed that older adults with more positive self-perceptions of aging reported less distress [9]. Research in Catalonia on frail older adults identified prelockdown depressive symptoms and engagement in leisure activities as important predictors of distress. Although self-efficacy was not directly measured, personal resources and coping strategies were found to be essential for reducing distress [23].

In addition to examining the well-established predictors of loneliness and psychological distress, this study introduces PS as a potential contributing factor to mental health outcomes in older adults. Although PS has been studied in other age groups [24,25], its impact on loneliness and psychological distress among older adults during periods of social isolation has remained under-researched. This gap in the literature presents an opportunity to explore the role of PS in older adults’ mental well-being. PS, defined as the voluntary pursuit of time alone that is both enjoyable and productive [26,27], may affect well-being during periods of extreme social distancing. PS typically involves voluntary social isolation, where a person may enjoy solitude without experiencing loneliness, just as one can feel lonely even when surrounded by others [28]. This concept highlights the distinction between being alone and feeling lonely, which is essential for understanding how different social dynamics affect mental health. Research on the effects of PS reveals a notably inconsistent and complex picture. Some studies suggest that enjoying solitude can reduce negative emotions and improve emotional well-being [29]. However, other research underscores that the impact of solitude on well-being is highly context-dependent and not straightforward. For older adults, certain dimensions of solitude—for example, feeling productive when alone—have been linked to greater life satisfaction and increased positive affect [30,31]. Conversely, PS is also frequently associated with feelings of loneliness, which can undermine subjective well-being [30]. This contradictory evidence illustrates a puzzling inconsistency—while PS might buffer some negative effects of social isolation, it does not consistently translate into better mental health outcomes. This phenomenon, termed the “preference for solitude paradox” [32], highlights that PS does not always provide the expected psychological benefits, especially during times of widespread isolation, such as global crises [27]. Overall, these mixed findings emphasize the urgent need for a deeper investigation into how and when PS influences mental health in older adults.

The empirical findings presented in the “Introduction” section highlight gaps in existing knowledge that will be addressed by answering the research aims.

First, this study explored personal and situational factors contributing to loneliness in older adults during COVID-19, extending regional findings to a national level. Second, it examined how pandemic-induced changes in social routines (eg, spending time alone or socializing with others), PS, and self-efficacy predicted psychological distress. Finally, the study investigated the mediating role of PS in the relationship between pandemic stressors and self-efficacy, focusing on its contribution to maintaining functionality during the pandemic.

## Methods

### Overview

This study used a cross-sectional, survey-based research design with a nonrandomized sampling method. The study design, data collection, and reporting followed the STROBE (Strengthening the Reporting of Observational Studies in Epidemiology) guidelines for cross-sectional studies [33].

### Participants

Participants were Croatian citizens of both genders, aged 65 years and older, living in Croatia. To define older adults, we used a cutoff age of 65 years [34]. The sample comprised 2053 participants.

As shown in Table 1, the sample was mostly composed of older adults, with a majority being female, residing in urban areas, and either married or widowed. More specifically, the median age of the participants was 74 (IQR 69-81) years. Regarding gender distribution, women were more represented in the sample (n=1324, 64.49%) than men (n=729, 35.51%). Most participants lived in urban areas (n=1585, 76.57%), while a smaller proportion lived in rural areas (n=468, 22.61%). In terms of marital status, about half of the participants were married (n=1000, 48.71%). A significant proportion were widowed for over 1 year (n=745, 36.29%), while smaller percentages were recently widowed (less than 3 months: n=65, 3.17%; 3 months to 1 year: n=56, 2.73%). In addition, 97 (4.73%) of participants had never been married, and 90 (4.38%) were divorced.

**Table 1.** Sociodemographic characteristics of participants in a cross-sectional study of older adults in Croatia during the COVID-19 pandemic (2021).

Variables	Descriptive statistic
Age (years), median (IQR)	74.0 (69-81)
Gender, n (%)	
Man	729 (35.51)
Women	1324 (64.49)
Place of residence, n (%)	
Urban	1585 (76.57)
Rural	468 (22.61)
Marital status, n (%)	
Married	1000 (48.71)
Divorced	90 (4.38)
Never married	97 (4.73)
Widowed (<3 months)	65 (3.17)
Widowed (3 months to 1 year)	56 (2.73)
Widowed (>1 year)	745 (36.29)

### Procedure

Participants were recruited from community settings using a snowball sampling technique [35,36]. Initially, health students from 6 Croatian cities (Zagreb, Split, Rijeka, Zadar, Dubrovnik, and Osijek), each guided by a university professor, were instructed to identify 6 individuals aged 65 years and older among their relatives, ensuring inclusion of both sexes. Eligible participants were community-dwelling older adults who were able to understand the study purpose and complete the questionnaire independently. Each recruited participant was then asked to invite up to 6 peers from their own social circles (friends or relatives) who met the same criteria. Data collection continued until the target sample size (n=2053) was achieved, calculated using the Cochrane formula for continuous data [37].

Before participation, potential respondents were informed about the study aims, the voluntary nature of participation, and the guarantee of anonymity and confidentiality. Anonymity of the respondents was ensured by placing each completed questionnaire in a sealed envelope. After filling out the questionnaires, respondents returned the sealed envelopes to the student. The students then delivered the envelopes to their supervising professor, who subsequently forwarded them to the principal investigator. The research was conducted between April and October 2021 in nonclinical, community-based environments (primarily private households).

### Measures

The following measures were used to collect data and address the research objectives:

#### Revised UCLA Loneliness Scale (Croatian Short-Form Version)

This 7-item scale measures loneliness as a general unidimensional construct, defined as an unpleasant emotional experience that arises when an individual’s needs for intimacy, love, and belonging are unmet. Participants rate each item on a 5-point Likert scale, ranging from 1 (not at all true of me) to 5 (completely true of me). The total score was calculated by averaging all item scores and dividing by the number of items [38]. The unidimensional structure and high reliability of the scale have been confirmed in both international and Croatian samples. In the Croatian sample, Cronbach α was 0.84 [39].

#### PS Scale (Croatian Adapted Version)

This 7-item questionnaire measures the degree of PS, with each item rated on a 5-point Likert scale. According to Burger [26], the scale demonstrated internal consistency (Cronbach *α*=0.70-0.73) and adequate validity for measuring PS. Further validation by Cramer and Lake [40] yielded similar internal consistency (Cronbach *α*=0.74-0.75) and test-retest reliability (*r*=0.76) over a 6- to 8-week interval. In the Croatian version, reliability coefficients ranged from Cronbach *α*=0.74-0.79 [41].

#### Clinical Outcomes in Routine Evaluation–Outcome Measure (CORE-OM; Croatian Version)

This instrument assesses psychological distress through 34 self-report items covering 4 dimensions: subjective well-being (4 items), problems or symptoms (12 items), daily functioning (12 items), and risky behaviors (6 items). These domains represent different aspects of psychological distress and dysfunction. Participants rate how frequently they experienced the described feelings during the past 2 weeks, on a scale from 0 (never) to 4 (almost always). Higher scores indicate greater psychological distress, with 8 positively worded items reverse-scored [42]. The Croatian version demonstrated high internal consistency (Cronbach *α*=0.92 [43]).

#### General Self-Efficacy Scale (GSE; Croatian Version)

This 10-item scale assesses an individual’s belief in their ability to handle new and challenging tasks across different contexts. Items are rated on a 5-point scale, from 1 (not at all true of me) to 5 (completely true of me). For example, the statement “I am confident that I can successfully handle unexpected situations” reflects general self-efficacy, with higher scores indicating stronger self-belief. The scale has demonstrated positive psychometric properties [44]. A review by Scholz et al [45] reported internal consistency coefficients across samples and countries ranging from 0.75 to 0.91. Evidence supports a unidimensional factor structure [46]. In the Croatian version, Cronbach α ranged from 0.74 to 0.85 [47].

#### Pandemic-Specific Stressors Questionnaire for Older Adults (PSQ-OA)

This questionnaire measures pandemic-specific stressors among older adults. It includes 8 statements rated on a 5-point scale from 0 (not at all) to 4 (extremely), where respondents assess their level of concern related to the pandemic. Exploratory factor analysis revealed 2 factors: social distancing behaviors (5 items) and exposure to infection (3 items). The scale demonstrated good reliability (Cronbach α=0.88 [19]).

#### General Data Questionnaire

This questionnaire collected demographic information, such as gender, age, place of residence, and marital status, as well as questions about time spent alone and social interactions. It also included items on neurological conditions (used as exclusion criteria) and questions about social behavior during quarantine.

### Statistical Analysis

Descriptive measures were first used to summarize demographic data, with frequencies and percentages calculated for categorical variables such as gender, marital status, time spent alone, socializing with others, and place of residence. Means and SDs were used for continuous variables such as age, loneliness, psychological distress, and self-efficacy. Hierarchical regression analysis was conducted in a stepwise manner to assess the contribution of various predictors to the dependent variables of loneliness and psychological distress. The predictors included demographic factors, psychological factors, and social behaviors during the lockdown. Model fit was assessed using *R*², *F* statistics, and *P* values. In addition, path analysis was used to examine the mediating contribution of PS (mediator) in the relationship between pandemic-specific stressors (social distancing behaviors and exposure to infection as predictors) and self-efficacy as the dependent variable. Path coefficients, SEs, and CIs were reported for direct, indirect, and total effects. The significance level was set at *P*<.05 for all tests.

### Use of Artificial Intelligence Tools

ChatGPT (OpenAI) was used only as a supportive technical tool in this study. Specifically, it was used to provide suggestions for improving the accuracy of English grammar and phrasing. The authors carefully verified, edited, and approved all artificial intelligence–generated content and take full responsibility for the final paper.

### Ethical Considerations

This study was conducted in accordance with the Declaration of Helsinki and was approved by 7 ethics committees (Table 2). All participants provided written informed consent before participation. One copy of the consent form was kept by the research participant, and a second was stored separately from the questionnaire to maintain anonymity. Data collection and privacy protection were conducted in accordance with the General Data Protection Regulation (2016/679). All data were anonymized before analysis, and no identifying information was collected, stored, or reported. Participants did not receive any financial or other compensation for their participation in the study. No images or additional materials contain identifiable information about study participants.

**Table 2.** List of ethical approvals for a cross-sectional study of older adults in Croatia during the COVID-19 pandemic (2021).

University Ethics Committee	Approval number	Date of approval
School of Medicine, University of Split	Class: 003-08/20-03/0005; registration number (reg no): 2181-198-03-04-20-0083	June 30, 2020
Faculty of Health Sciences, University of Split	Class: 001-01/20-01/0009; reg no: 2181-228-07-20-0005	November 13, 2020
Faculty of Health Studies, University of Rijeka	Class: 602-04/21-01/85; reg no: 2170-57-5-07-1-21-1	March 13, 2021
University of Dubrovnik	Reg no: 524/21	April 30, 2021
University of Applied Health Sciences, Zagreb	Class: 602-04/21-18/222; reg no: 251-379-10-21-02	May 18, 2021
University of Zadar	Class: 114-06/21-01/15; reg no: 2198-1-79-62-21-02	May 25, 2021
Faculty of Dental Medicine and Health, Josip Juraj Strossmayer University of Osijek	Class: 602-04/20-08/02; reg no: 2158/97-97-07-21-08	June 2, 2021

## Results

As outlined in the first research aim, Table 3 shows the predictive value of sociodemographic and psychological factors, as well as PS and pandemic-related stressors, on loneliness in older adults during the COVID-19 pandemic. The first step in the model included an initial set of sociodemographic predictors, explaining 5.4% of the variance in loneliness, suggesting that while the model provides a small but statistically significant contribution, much of the variance remains unexplained. In the second step, psychological predictors were introduced, significantly improving the model’s explanatory power. The increase in *R*^2^ (from 0.054 to 0.294) indicates that these new variables accounted for an additional 24% of the variance. This represents the largest improvement across all steps. The third step introduced pandemic-specific stressors, leading to a modest yet statistically significant improvement in model accuracy (*R*^2^=0.302; *P*<.001), contributing an additional 0.8%. The final step added PS as an additional predictor, resulting in a small but significant increase in explained variance (*R*^2^=0.307; *P*<.001), accounting for only 0.5%.

**Table 3.** Hierarchical regression analysis of demographic and psychological variables, pandemic-specific stressors, and preference for solitude as predictors of loneliness among older adults during the COVID-19 pandemic (2021).

Predictor	Step 1 (B^a^)	Step 2 (B)^a^	Step 3 (B)^a^	Step 4 (B)^a^
Demographic variable				
Age	–0.002	–0.006^b^	–0.006^b^	–0.005^b^
Gender (women)	–0.023	–0.072^b^	–0.085^b^	–0.081^b^
Marital status				
Divorced	0.219^b^	0.048	0.067	0.026
Never married	0.676^c^	0.407^c^	0.417^c^	0.390^c^
Widowed (<3 months)	0.581^c^	0.324^c^	0.321^c^	0.292^c^
Widowed (3 months-1 year)	0.416^c^	0.128	0.142	0.137
Widowed (>1 year)	0.274^c^	0.189^c^	0.191^c^	0.180^c^
Place of residence (rural)	–0.154^c^	–0.108^c^	–0.103^c^	–0.100^b^
Psychological variable (CORE^d^)				
Functionality	—	–0.041^c^	–0.039^c^	–0.037^c^
Problems	—	0.023^c^	0.020^c^	0.020^c^
Subjective well-being	—	–0.014	–0.012	–0.011
Risky behaviors	—	–5.49×10-4	–0.002	–0.001
PSQ^e^				
Social distancing	—	—	0.012^c^	0.014^c^
Exposure to infection	—	—	0.009	0.008
Preference for solitude				0.011^c^
R^f^	0.232	0.542	0.550	0.554
*R*^2g^	0.054	0.294	0.302	0.307
RMSE^h^	0.826	0.714	0.710	0.708
*F*-statistic	14.472	70.78	62.99	60.256
*P* value	<.001	<.001	<.001	<.001

a B: unstandardized regression coefficient.

b *P*<.05.

c P<.01.

d CORE: Clinical Outcomes in Routine Evaluation.

e PSQ: pandemic-specific stressor.

f R: correlation coefficient.

g *R*²: coefficient of determination.

h RMSE: root mean square error.

Regarding the individual contribution of each predictor in the final step of the regression analysis, age was found to have a small but significant negative relationship with loneliness (unstandardized regression coefficient [B]=−0.005; *P*=.05), indicating that individuals in older groups experienced lower levels of loneliness. Gender also emerged as a significant factor, with women reporting lower loneliness compared with men (B=−0.081; *P*<.02). Marital status played an important role in loneliness levels, where living alone was significantly associated with increased loneliness (B=0.390; *P*<.001), as was being recently widowed (B=0.292; *P*=.002 and B=0.180; *P*<.001, respectively). A significant association was also found between place of residence and loneliness, with individuals living in rural areas reporting lower levels of loneliness compared with those in urban settings (B=−0.100; *P*=.008).

Psychological factors were strongly related to loneliness, where higher levels of psychological problems were significantly associated with greater loneliness (B=0.020; *P*<.001), while higher functionality was related to lower loneliness (B=−0.037; *P*<.001).

Further, increased social distancing was significantly associated with higher loneliness (B=0.014; *P*<.001), suggesting that restrictions on social interactions contributed to feelings of isolation.

Finally, PS was significantly related to higher loneliness (B=0.011; *P*<.001), suggesting that while some individuals may choose solitude, it can still contribute to feelings of loneliness (Table 3).

Furthermore, as stated in the second research aim, the study examined the contribution of lockdown activities (spending time alone or with others), PS, and self-efficacy to psychological distress levels among older adults. The hierarchical regression analysis assessed how various social and psychological factors during lockdown predicted psychological distress, as measured by Clinical Outcomes in Routine Evaluation total scores (Table 4).

**Table 4.** Hierarchical regression analysis of frequency of socialization, preference for solitude, and self-efficacy as predictors of psychological distress among older adults during the COVID-19 pandemic (2021).

Predictor	Step 1 (B)^a^	Step 2 (B)^a^	Step 3 (B)^a^
Time spent alone			
Being alone (only at night)	–1.656	–1.460	–1.827
Being alone (only during the day)	–1.820	–2.510	–3.705
Being alone (> half a day)	0.961	1.039	1.006
Being alone (several hours a day)	–5.173^b^	–4.908^b^	–3.509^b^
Being alone (never longer than 1 hour)	–4.613^b^	–3.946^b^	–2.968^c^
Socializing with others during lockdown			
Close friends (>1 week)	–0.915	–1.076	–1.054
Close friends (at least once a week)	–0.659	–0.701	–1.536
Close friends (2‐3 times a month)	–0.174	–0.325	–1.631
Close friends (once per month)	0.822	0.673	–1.141
Close friends (once in 6 months)	–0.457	–0.813	–5.156^c^
Close friends (very rare)	5.172^b^	5.058^b^	0.845
Acquaintances (multiple times per week)	1.042	1.050	2.496
Acquaintances (at least once a week)	2.432	2.471	4.321^b^
Acquaintances (2‐3 times a month)	2.444	2.154	4.543^b^
Acquaintances (once per month)	3.845^c^	3.506^c^	4.357^b^
Acquaintances (once in 6 months)	1.135	0.984	4.163^c^
Socializing with acquaintances (very rare)	7.069^b^	6.750^b^	6.690^b^
Last visit			
Family member (1 month ago)	0.202	0.412	3.895
Family member (1 week ago)	–1.604	–1.371	1.359
Family member (yesterday or today)	–3.396	–3.093	0.481
Another relative	4.083^b^	3.950^b^	3.962^b^
Friends	0.335	0.061	1.127
Nonfamily member	1.374	1.213	3.353^c^
Preference for solitude	—	1.593^b^	2.403^b^
Self-efficacy			–1.066^b^
R^d^	0.299	0.308	0.555
*R*^2^^e^	0.089	0.095	0.308
RMSE^f^	17.059	17.011	14.872
*F* statistic	8.631	8.835	36.156
*P* value	<.001	<.001	<.001

a B: unstandardized regression coefficient.

b *P*<.01.

c *P*<.05.

d R: correlation coefficient.

e R²: coefficient of determination.

f RMSE: root mean square error.

The results consistently showed an improvement in the model’s ability to explain psychological distress among older adults during the lockdown across the different regression steps. In Step 1, which included variables related to time spent alone and the frequency of social interactions, the model accounted for 8.9% of the variance. Step 2 introduced PS, slightly improving the model’s explanatory power to 9.5%. Although this was a modest increase, it indicated that preference toward solitude contributed uniquely to psychological outcomes. The final model (Step 3), which introduced self-efficacy, led to a substantial increase in explanatory power, explaining 30.8% of the variance and underscoring the pivotal role of personal psychological resources in predicting distress levels during the lockdown period. Self-efficacy showed a strong negative relationship with psychological distress (B=−1.066; *P*<.001), indicating that older adults who believe in their capacity to handle challenges tended to report significantly lower levels of distress. Despite the prevalence of self-efficacy, several other predictors remained significant. Specifically, being alone for several hours per day and never being alone longer than an hour were both significantly associated with lower distress (B=−3.509; *P*=.003 and B=−2.968; *P*=.01, respectively), suggesting that a short amount of time spent alone may have protective effects on the mental health of older adults. On the contrary, rare contacts with close friends, once every 6 months, were associated with lower distress (B=−5.156; *P*=.02), highlighting the emotional importance of regular, meaningful friendships. Further, both frequent and infrequent interactions with acquaintances (weekly to rarely) were consistently associated with higher distress (B value varied from 4.163 to 6.690; all *P*<.05), implying that interactions lacking emotional closeness or confidence may not be beneficial and could even contribute to distress. Visits from distant family members (B=3.962; *P*<.001) and nonfamily members (B=3.353; *P*=.04) were also significant predictors of distress, suggesting these social contacts may have been stress-inducing under lockdown conditions. Finally, PS remained a significant positive predictor of distress (B=2.403; *P*<.001), suggesting that individuals who generally enjoy being alone may have still experienced emotional loneliness during enforced isolation.

**Figure 1.**
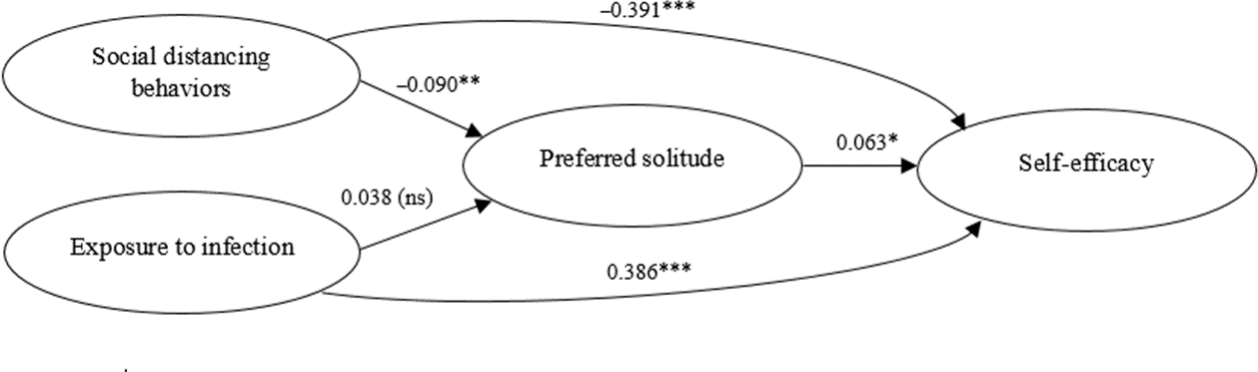
Path model showing direct and indirect effects of pandemic-specific stressors on self-efficacy, with preference for solitude as a mediator. *P<.05; **P<.01; ***P<.001; ns: nonsignificant.

The third research aim investigated the mediating role of PS in the relationship between pandemic stressors (social distancing behaviors and exposure to infection) and self-efficacy among older adults. Social distancing behaviors and exposure to infection were included as predictor variables in the path analysis, while self-efficacy was considered the outcome variable. PS, or an individual’s tendency to seek time alone, was examined as a mediator that may either buffer or amplify the effects of these stressors on self-efficacy (Table 5; Figure 1).

**Table 5.** Path analysis of the mediating role of preference for solitude in the relationship between pandemic-specific stressors and self-efficacy among older adults during the COVID-19 pandemic (2021). Criterion: self-efficacy; Mediator: preference for solitude.

Path and effect	Social distancing behaviors as a predictor			Exposure to infection as a predictor			Path coefficients		
	Direct effects	Indirect effects	Total effects	Direct effects	Indirect effects	Total effects	Social distancing behaviors and preference for solitude	Exposure to infection and preference for solitude	Preference for solitude and self-efficacy
β^a^ (95% CI)	–0.391 (–0.477 to –0.303)	–0.006 (–0.016 to –0.000)	–0.396 (–0.487 to –0.312)	0.386 (–0.477 to –0.303)	0.002 (–0.003 to 0.016)	0.388 (0.252 to 0.534)	–0.090 (–0.161 to –0.023)	0.038 (–0.067 to 0.158)	0.063 (–0.002 to 0.123)
SE	0.043	0.003	0.043	0.069	0.003	0.069	0.033	0.052	0.029
*P* value	<.001	.09	<.001	<.001	.48	<.001	.006	.46	.03

a β: standardized regression coefficient

Social distancing behaviors were negatively associated with self-efficacy, showing a strong and significant direct effect (*β*=−.391; *P*<.001). The indirect pathway through PS was small and not significant (*β*=−.006; *P*=.09), resulting in a total negative effect of social distancing on self-efficacy (*β*=−.396; *P*<.001). By contrast, exposure to infection demonstrated a strong positive direct association with self-efficacy (*β*=.386; *P*<.001). Its indirect effect through PS was negligible and nonsignificant (*β*=.002; *P*=.48), yielding a total positive effect (*β*=.388; *P*<.001). Furthermore, PS was positively related to self-efficacy (*β*=.063; *P*=.03), suggesting that individuals with a higher PS reported slightly greater levels of self-efficacy. The path from social distancing to PS was significant and negative (*β*=−.090; *P*=.006), whereas the path from exposure to infection to PS was nonsignificant (*β*=.038; *P*=.46). Overall, the findings indicate that while pandemic stressors exerted significant direct effects on self-efficacy, PS contributed only a small positive direct effect and did not act as a strong mediator in these relationships.

## Discussion

### Principal Findings

This study aimed to evaluate how sociodemographic and psychological factors, along with exposure to pandemic-related stressors and PS, were associated with loneliness among older adults during the COVID-19 pandemic. Second, it explored how pandemic-induced changes in social routines, self-efficacy, and PS predicted levels of psychological distress in this population. Finally, it examined whether PS mediated the relationship between pandemic stressors and self-efficacy.

The findings of this study closely mirrored those of the earlier pilot study [19], highlighting consistent risk factors for loneliness among older adults during the COVID-19 pandemic. Similar to previous international research, predictors such as living alone [11], widowhood [48], psychological distress [2], and social isolation [49] were reaffirmed as significant contributors to loneliness. Older adults and women reported lower levels of loneliness, possibly due to caregiving roles and strong intergenerational ties that are common in Croatian society. This contrasts with previous research showing that older women are more vulnerable to loneliness [17,50]. In our study, men appeared more at risk, likely due to weaker social or family engagement. Similarly, rural residents reported lower loneliness than urban residents, opposing earlier findings [14], possibly due to rural self-reliance and reduced dependence on frequent social interaction. This study also showed lower loneliness among older adults, contrary to some previous studies [51,52]. This may reflect cultural or contextual factors that help explain differences observed in the Croatian sample, as identified in earlier research. For example, older adults in Croatia often benefit from strong family ties and intergenerational support [53]. They may also be more resilient due to past experiences with hardship, such as war or economic instability [54], or may feel more comfortable with smaller, stable social networks that are less affected by crises [55].

This study also examined the role of PS in older adults’ mental health and self-efficacy during the COVID-19 pandemic, revealing a complex relationship. Previous studies have shown that PS can support autonomy, emotional regulation, and self-reflection [56,57]. However, recent research [27,58], including this study, suggests that these benefits are context dependent. Our findings show that a higher PS is associated with increased loneliness and psychological distress during enforced isolation. This aligns with previous research suggesting that even voluntary solitude can become emotionally burdensome under restrictive conditions. This tension highlights the need to distinguish between voluntary solitude sought for restoration and involuntary solitude experienced during social constraints (eg, pandemic lockdowns [27]). Theoretically, PS may be protective only when individuals have the agency to choose when and how they are alone. When external circumstances remove that choice, the same preference may leave individuals more vulnerable, especially if it co-occurs with limited social support [32].

PS showed a small but statistically significant association with older adults’ well-being, accounting for only limited variance in loneliness and self-efficacy. In contrast, self-efficacy emerged as a stronger and more consistent predictor, reinforcing its role as an important psychological resource in later life. This finding is consistent with previous research highlighting the protective effects of self-efficacy against loneliness [59]. It may help buffer distress through various mechanisms, including enhanced coping, reduced helplessness, and a greater sense of control—factors emphasized in Bandura’s social cognitive theory [60]. While PS may offer certain benefits, its effects appear modest compared with the pronounced impact of self-efficacy. Therefore, psychosocial interventions may include elements that support PS but with realistic expectations of its overall influence. Intervention programs for older adults should prioritize the development of self-efficacy through mastery experiences, meaningful engagement, coping skills training, and supportive social environments. Such approaches are more likely to reduce loneliness and improve emotional well-being in later life.

The path analysis showed that pandemic-related experiences, including social distancing and infection exposure, had the strongest impact on self-efficacy. In contrast, PS had only a minor effect. No studies directly link PS with self-efficacy among older adults in the context of the pandemic. However, existing research [61-64] highlights the dominant role of situational factors such as lockdowns and isolation in shaping psychological outcomes, including self-efficacy. This supports our finding that dispositional factors such as PS are less central in explaining self-efficacy during the pandemic.

Another hierarchical regression analysis in this study revealed several significant predictors of psychological distress (measured by Clinical Outcomes in Routine Evaluation scores). The findings indicate that lower self-efficacy is an important risk factor for psychological distress among older adults, reinforcing existing evidence of its protective role in mental health [61-64]. PS also emerged as a significant contributor to distress, even after accounting for self-efficacy and social behaviors. This finding indicates that solitude may function as a vulnerability factor in this context [27,32]. Moderate amounts of time spent alone were associated with lower distress, suggesting a potential restorative value of solitude. In contrast, limited or superficial social interactions—particularly with acquaintances—were linked to higher levels of psychological strain. This pattern underscores the importance of relationship quality over quantity during periods of social disruption. Our findings resonate with previous research showing that emotionally close ties offer more effective emotional support than weaker social connections, particularly in times of crisis [65,66]. Weak ties, by contrast, may fail to provide meaningful support and, in some cases, may even intensify feelings of loneliness or stress [67]. Interventions aimed at reducing psychological distress in older populations should not simply encourage more social interaction but should instead prioritize the development of deeper, emotionally meaningful connections.

This study, based on a large national sample of older adults, makes a valuable scientific contribution. It highlights important risk and protective factors for mental health and self-efficacy in later life during a global crisis. One of the main contributions lies in examining the underexplored construct of PS, which sheds light on the complex role PS plays in shaping mental health outcomes during enforced social isolation. Although often beneficial, a PS may increase loneliness and distress during crises, particularly when social autonomy is limited. The study also reinforces the importance of psychological well-being and social connectedness as central to maintaining mental health and self-efficacy in older populations during disruptive events.

In practical terms, the findings can inform public health policies and guide targeted interventions aimed at preventing mental health decline among older adults during future pandemics or similar emergencies. Recognizing that loneliness and distress can arise even in individuals who prefer solitude is crucial, as it highlights the need for nuanced strategies that respect individual preferences while maintaining strong social support systems. In addition, the role of self-efficacy as a buffer against distress is significant, highlighting the importance of empowering older adults with clear information, accessible support services, and opportunities to maintain independence during crises. Interventions tailored to rural and urban differences and designed to be sensitive to gender- and age-specific vulnerabilities could further improve outcomes.

### Limitations

Despite its strengths, the study has several limitations. The use of snowball sampling was efficient in reaching a large number of older adults across multiple Croatian cities. However, it may have introduced selection bias and limited the representativeness of the findings. Participants were likely drawn from similar networks, resulting in a more homogeneous sample in terms of socioeconomic status, education, health awareness, and other factors. This may restrict the generalizability of the results, particularly to more vulnerable or socially isolated older adults who may have been underrepresented. This sampling method was chosen due to the lockdown conditions at the time of data collection, as epidemiological measures severely limited older adults’ ability to leave their homes. Snowball sampling was the only feasible way to reach them under those circumstances. Furthermore, the cross-sectional design prevents causal inferences regarding the observed relationships. Future research should consider using probability-based sampling to enhance representativeness, especially among marginalized or hard-to-reach populations. In addition, future research should use longitudinal designs, as they can help test causality by establishing temporal order, clarifying whether changes in solitude precede changes in psychological distress or vice versa. Such designs allow researchers to track how variables evolve and examine whether shifts in one predict shifts in another. By controlling for baseline levels and assessing potential mediators or moderators, such as social support or health status, longitudinal studies can provide deeper insight into the mechanisms underlying these relationships and offer stronger causal inferences. Finally, qualitative approaches may offer deeper insights into how older adults experience and navigate solitude, stress, and resilience during crises, supporting the development of more targeted mental health interventions.

### Conclusion

This study examined how sociodemographic, psychological, and pandemic-related factors affected loneliness and distress in older adults during COVID-19. It also explored whether PS mediated the relationship between pandemic stressors and self-efficacy. Most of these factors affected loneliness, accounting for about one-third of its variance. This finding supports and extends previous regional findings using a nationally representative sample. Changes in social routines, PS, and self-efficacy also predicted psychological distress, explaining roughly one-third of its variance. Spending a moderate amount of time alone was linked to lower distress, while limited or superficial social interactions increased it. Self-efficacy emerged as a strong protective factor against psychological distress. Although PS had only a small mediating role between pandemic stressors and self-efficacy, it still affected mental health. Overall, situational factors had the greatest impact on mental well-being. Future interventions should focus on strengthening self-efficacy and promoting meaningful social connections among older adults during times of crisis.

## Abbreviations

PS: preference for solitude
STROBE: Strengthening the Reporting of Observational Studies in Epidemiology
